# Material Substrate Physical Properties Control Pseudomonas aeruginosa Biofilm Architecture

**DOI:** 10.1128/mbio.03518-22

**Published:** 2023-02-14

**Authors:** Alice Cont, Joseph Vermeil, Alexandre Persat

**Affiliations:** a Institute of Bioengineering and Global Health Institute, School of Life Sciences, Ecole Polytechnique Fédérale de Lausanne, Lausanne, Switzerland; University of Michigan-Ann Arbor

**Keywords:** biofilms, mechanics, twitching motilty

## Abstract

In the wild, bacteria are most frequently found in the form of multicellular structures called biofilms. Biofilms grow at the surface of abiotic and living materials with wide-ranging mechanical properties. The opportunistic pathogen Pseudomonas aeruginosa forms biofilms on indwelling medical devices and on soft tissues, including burn wounds and the airway mucosa. Despite the critical role of substrates in the foundation of biofilms, we still lack a clear understanding of how material mechanics regulate their architecture and the physiology of resident bacteria. Here, we demonstrate that physical properties of hydrogel material substrates define P. aeruginosa biofilm architecture. We show that hydrogel mesh size regulates twitching motility, a surface exploration mechanism priming biofilms, ultimately controlling the organization of single cells in the multicellular community. The resulting architectural transitions increase P. aeruginosa’s tolerance to colistin, a last-resort antibiotic. In addition, mechanical regulation of twitching motility affects P. aeruginosa clonal lineages, so that biofilms are more mixed on relatively denser materials. Our results thereby establish material properties as a factor that dramatically affects biofilm architecture, antibiotic efficacy, and evolution of the resident population.

## INTRODUCTION

Bacteria preferentially colonize surface environments as multicellular communities called biofilms (1). Biofilms form when bacteria attach to surfaces and subsequently divide while embedding themselves in a self-secreted matrix (2, 3). Cells residing in biofilms have selective advantages compared to their planktonic counterpart. For example, the matrix mechanical properties not only provide cohesion but also physically protect cells against physical and predatory challenges such as flow and grazing (3). The biofilm lifestyle also confers protection against chemical stressors such as antimicrobials (4). As a result, biofilm-dwelling bacterial populations are overall more tolerant of antibiotic treatment. The resilience of biofilms against antibiotic treatment has both biological and physical causes (4, 5). Biofilm residents undergo metabolic adaptation that decreases their drug sensitivity. On the physical side, the presence of the matrix and the three-dimensional cellular arrangement within a biofilm can reduce the penetration of molecular compounds to its core (4, 5). As a result, biofilm architecture can critically regulate bacterial tolerance to antibiotic treatment.

Due to their resilience, biofilms are a common cause of chronic, persistent infections (6, 7). Biofilms of the opportunistic pathogen Pseudomonas aeruginosa frequently cause burn wound infections and chronic airway infections, particularly in individuals with cystic fibrosis. To form biofilms, P. aeruginosa explores surfaces using surface-specific twitching motility powered by type IV pili (8). During this process, single cells aggregate and initiate a collective growth phase that results in biofilm maturation. P. aeruginosa biofilms frequently grow in contact with soft host tissue, including cells and extracellular matrices such as mucus (5). The mechanical properties of these materials are quite different from the ones P. aeruginosa encounters in traditional biofilm assays in the lab, which employ much stiffer materials, including glass and hard plastics. As a result, our knowledge of biofilm formation on realistic soft substrates remains limited.

Mechanics play an important role during the development of a biofilm (9, 10). When cells grow to form a biofilm, they exert forces on their elastic matrix that ultimately generate internal mechanical stresses. The buildup of internal stress has an impact on biofilm morphogenesis, causing instabilities such as buckling or wrinkling (11). When growing on soft surfaces, these instabilities cause material substrates to deform, be they hydrogels or host epithelial tissues (12). Material surface physicochemical properties such as topography, chemistry, charge, and hydrophobicity have an effect on the adhesion of single bacteria (13–18). However, despite host-associated biofilms ubiquitously forming on soft surfaces of various rigidities, the mechanisms by which mechanical properties of a substrate affect the structure and organization of a biofilm have been neglected. Explorations of bacterial physiology on polydimethylsiloxane (PDMS) and hydrogels indicate that material mechanics may mediate biofilm formation (19–21). Despite the conclusions contrasting across materials, this suggests that substrate rigidity could play a role in bacterial physiology and biofilm formation.

Given the importance of surface sensing in P. aeruginosa in twitching motility (22) and in the onset of biofilm formation (23), we hypothesized that the mechanical properties of material substrates influence the process of biofilm formation. We tested this hypothesis by employing synthetic hydrogels with finely tunable mechanical properties. We found that substrate material properties have a profound impact on biofilm architecture. We could attribute these differences to the initial exploratory phase: P. aeruginosa’s surface motility differs between materials with different mesh sizes but with identical stiffness and chemistry. As a result, biofilm populations on denser hydrogels form shallower biofilms that are more sensitive to the last-resort antibiotic colistin. In addition, modulation of twitching motility by substrate material properties affects the spatial organization of different bacterial lineages, which has a potential impact on social interactions in heterogenous biofilms.

## RESULTS

### P. aeruginosa biofilm architecture depends on substrate material properties.

**FIG 1:**
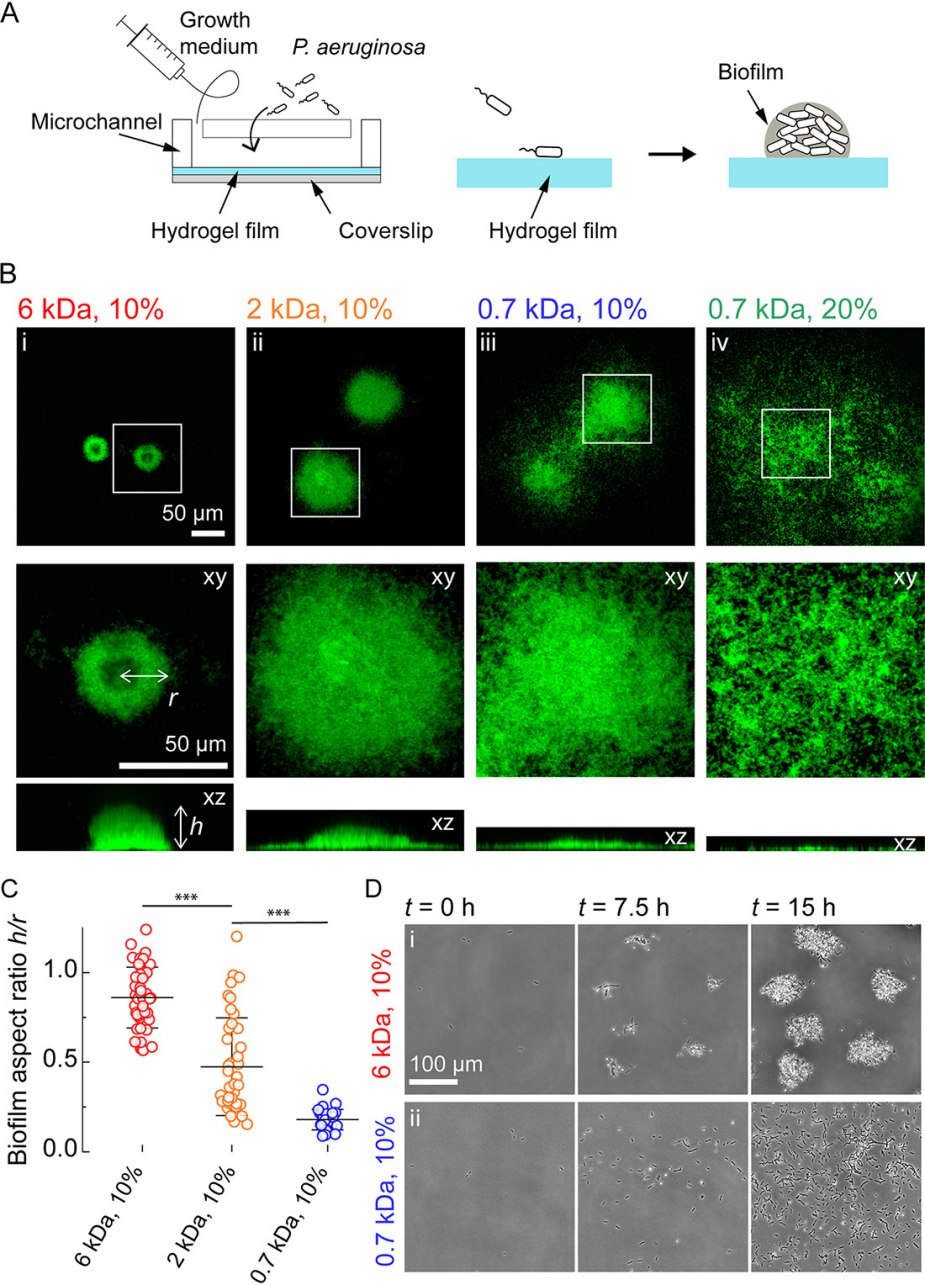
Hydrogel elastic substrates regulate P. aeruginosa biofilm architecture. (A) Illustration of experimental setup in which we generate thin poly(ethylene glycol) (PEG) hydrogel films at the bottom surface of microchannels. These devices allow us to study biofilm formation on soft materials at high resolution. (B) In-plane and cross-sectional confocal visualizations show different architectures of P. aeruginosa biofilms grown on hydrogels with different chain lengths (molecular weight [MW]) and concentrations of poly(ethylene glycol) diacrylate (PEGDA) precursors. For panel i, MW = 6,000 Da, 10% wt/vol; for panel ii, MW = 2,000 Da, 10% wt/vol; for panel iii, MW = 700 Da, 10% wt/vol; and for panel iv, MW = 700 Da, 20% wt/wt. (C) Quantification of biofilm height (*h*) and radius (*r*) on different hydrogels shows an increase in aspect ratio *h*/*r* with increasing chain length. Each circle corresponds to one colony; black bars represent means and standard deviation across all values. Colonies were selected from stacks acquired for three biological replicates. Statistics performed by one-way analysis of variance (ANOVA), followed by a *post hoc* Tukey test if the null hypothesis was rejected. Differences between the gels were statistically significant (*P < *0.001). Numerical values can be found in Table S2 at https://doi.org/10.5281/zenodo.7585216. (D) Time-lapse visualization indicates that hydrogels modulate P. aeruginosa biofilm architecture by regulating initial steps of biogenesis. (i) Biofilms form by division and local attachment in the vicinity of founder cells on long-chain PEGDA hydrogels. (ii) Single cells explore the surface of short-chain PEGDA hydrogels to form biofilms by aggregation.

To investigate the link between substrate mechanics and P. aeruginosa biofilm formation, we exploited poly(ethylene glycol) diacrylate (PEGDA) hydrogels. These synthetic polymeric networks are optically clear, are biocompatible, have homogeneous mechanical properties, and are relatively soft, thereby matching properties of living tissues (24). Moreover, PEGDA hydrogels are mechanically tunable: their elastic moduli can be readily manipulated by controlling cross-linking by modulating the concentration and/or the molecular weight of the precursors (25). To investigate the contributions of material properties on biofilm formation, we screened an assortment of PEGDA hydrogels cross-linked from prepolymer of different chain length (700 to 6,000 Da) and concentrations (10% to 30% wt/vol). We generated thin hydrogels films (~50 μm) at the bottom surface of microfluidic channels. These hydrogels were sufficiently thin to accommodate high-resolution confocal imaging. We seeded constitutively fluorescent P. aeruginosa strains at the surface of hydrogels and subsequently initiated biofilm growth under flow (Fig. 1A).

P. aeruginosa successfully colonized the surface of all hydrogels by forming biofilms. However, we observed differences in colonization patterns across conditions (Fig. 1B; Fig. S1). On hydrogels with relatively long and dilute polyethylene glycol (PEG), P. aeruginosa predominantly formed well defined isolated biofilms (Fig. 1B, panel i; Fig. S1A, panel i). These biofilms grew into tall structures with large height-to-width aspect ratios. In comparison, P. aeruginosa populations appeared to colonize more uniformly the surface of hydrogels as molecular weight decreased (Fig. 1B, panels i to iii) or PEGDA concentration increased (Fig. 1B, panels iii and iv; Fig. S1A, panels ii and iii). These biofilms were shallower, only slightly extending in the depth of the channel, normal to the surface. In the most extreme case of a 20% PEGDA at 700 Da, P. aeruginosa colonized the surface almost as a monolayer, making it difficult to distinguish biofilm colonies. In other words, decreasing the polymer chain length and increasing concentration promotes the transition from compact dome-shaped structures with near unity aspect ratio to flat and spread-out biofilms with relatively lower aspect ratio (Fig. 1C; Fig. S1B).

10.1128/mbio.03518-22.1 FIG S1 Hydrogel substrates regulate P. aeruginosa biofilm architecture. (A) In-plane and cross-sectional confocal visualizations show different architectures of P. aeruginosa biofilms grown on hydrogels with different molecular weights (MWs) and concentrations of poly(ethylene glycol) diacrylate (PEGDA) precursors. For panel i, MW = 3,400 Da, 10% wt/vol; for panel ii, MW = 6,000 Da, 20% wt; and for panel iii, MW = 6,000 Da, 30% wt. (B) Quantification of biofilm aspect ratio (*h*/*r*) on different hydrogels. Each circle corresponds to one colony, and black bars represent means and standard deviation across all values. The data points for the gels 6 kDa, 10%; 2 kDa, 10%; and 0.7 kDa, 10% are the same as those presented in Fig. 1C. The colonies for the other gels were selected from stacks acquired for one biological replicate. (C) In-plane and cross-sectional confocal visualizations show that biofilms look alike for a *ΔpilA* mutant on hydrogels with different composition, while differences in morphologies are still observable in biofilms of a *ΔfliC* mutant. *ΔpilA* and *ΔfliC* mutants constitutively express mScarlet. Download FIG S1, PNG file, 2.3 MB. Copyright © 2023 Cont et al. 2023 Cont et al. https://creativecommons.org/licenses/by/4.0/ This content is distributed under the terms of the Creative Commons Attribution 4.0 International license.

The stiffness of substrate materials can influence P. aeruginosa adhesion (21). We therefore anticipated that a mechanism associated with bacterial attachment and material rigidity could regulate biofilm biogenesis. We were, however, surprised that biofilms adopted strikingly different architectures on PEGDA hydrogels with nearly identical Young’s moduli (Fig. 1B; Table S1). We therefore subsequently investigated the mechanisms by which material properties regulate biofilm architecture. Time-lapse visualizations revealed noticeable differences between materials at the early stages of biofilm formation. Microcolonies rapidly appeared in the first few hours of growth on PEGDA hydrogels with high molecular weight (Fig. 1D, panel i; Movie S1). On shorter-chain PEGDA, we observed most P. aeruginosa cells exploring the surface as they grew and divided, leading to a more uniform distribution on the surface (Fig. 1D, panel ii; Movie S1). While this mechanism tends to decrease local cell density near founder cells, it still promotes bacterial aggregation on longer time scales, allowing the development of multicellular structures. These visualizations suggest that distinct hydrogels control biofilm architecture by regulating initial surface exploration. We therefore investigated how material properties could affect surface motility.

10.1128/mbio.03518-22.7 MOVIE S1 Time-lapse visualization of P. aeruginosa biofilm formation on PEGDA hydrogels with similar modulus (6 kDa, 10% [wt/vol] on the left, 0.7 kDa, 10% [wt/vol] on the right). Time is in h:min. Download Movie S1, AVI file, 11.4 MB. Copyright © 2023 Cont et al. 2023 Cont et al. https://creativecommons.org/licenses/by/4.0/ This content is distributed under the terms of the Creative Commons Attribution 4.0 International license.

10.1128/mbio.03518-22.10 TABLE S1 Summary of mechanical properties of PEGDA hydrogels. *, differences between 6 kDa, 10% and 3.4 kDa, 10% (*P* < 0.001); between 3.4 kDa, 10% and 2 kDa, 10% (*P* < 0.01); and between 2 kDa, 10% and 0.7 kDa, 10% (*P* < 0.05) are statistically significant (one-way analysis of variance [ANOVA], followed by a *post hoc* Tukey test). Download Table S1, DOCX file, 0.01 MB. Copyright © 2023 Cont et al. 2023 Cont et al. https://creativecommons.org/licenses/by/4.0/ This content is distributed under the terms of the Creative Commons Attribution 4.0 International license.

### Modulation of twitching motility via hydrogel substrate physical properties.

To nucleate biofilms, P. aeruginosa first attaches to and navigates on surfaces using twitching motility, which ultimately promotes aggregation (26). Long and thin retractile protein filaments called type IV pili (T4Ps) power twitching motility. T4Ps propel single cells forward by successively extending, attaching to the surface, and retracting (27). How substrate mechanical properties regulate twitching motility remains unclear, but theory predicts that cells move more efficiently on stiffer materials (28). Driven by our observations of early biofilm formation, we hypothesized that material properties regulate twitching motility, thereby leading to the different biofilm architectures. Consistent with this scenario, we found that a *ΔpilTU* deletion mutant that lacks type IV pili retraction machinery formed biofilms on shorter-chain PEGDA gels that resemble the ones formed by wild-type (WT) cells on long PEGDA gels (Movie S2). We confirmed that the observed morphologies are T4P-dependent and flagellum-independent. To achieve this, we grew biofilms of a *ΔpilA* mutant that lacks T4Ps. *ΔpilA* biofilms grew with dome-like morphologies irrespective of the hydrogel composition (Fig. S1C, panels i and ii). In contrast, a *ΔfliC* mutant that lacks flagella showed the same biofilm phenotype as WT (Fig. S1C, panels iii and iv).

10.1128/mbio.03518-22.8 MOVIE S2 Time-lapse visualization of P. aeruginosa biofilm formation on PEGDA hydrogels with similar modulus (6 kDa, 10% [wt/vol] on the left; and 0.7 kDa, 10% [wt/vol] in the center and on the right). PAO1 is in the left and central panels, and PAO1 ΔpilTU is in the right panel. Time is in h:min. Download Movie S2, AVI file, 12.0 MB. Copyright © 2023 Cont et al. 2023 Cont et al. https://creativecommons.org/licenses/by/4.0/ This content is distributed under the terms of the Creative Commons Attribution 4.0 International license.

**FIG 2:**
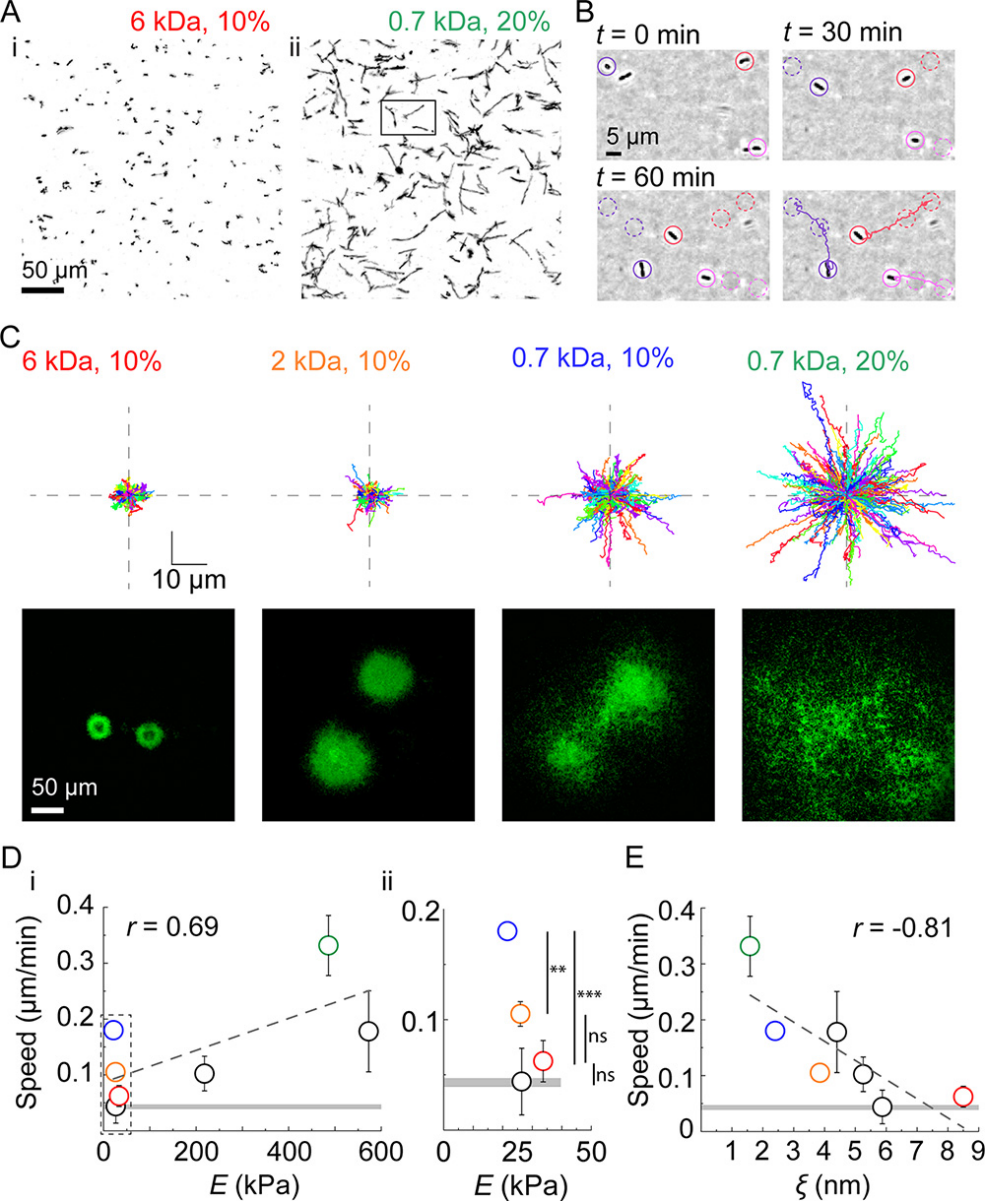
Hydrogel mesh size modulates biofilm architecture by regulating twitching motility. (A) Cumulative surface coverage of bacterial trajectories during the first hour of contact with the indicated poly (ethylene glycol) diacrylate (PEGDA) gel. Black signal corresponds to bacterial, white corresponds to unexplored surface. (B) Time-lapse visualization of P. aeruginosa twitching on the surface of PEGDA 700 Da at 20% and respective trajectories (from selection in panel ii in panel A). (C) Trajectories of 300 randomly selected cells (one replicate for illustrative purposes) on four different hydrogels and resulting biofilm morphology after 40 h of growth (reshow of Fig. 1). All trajectories start from the center of the graph. (D) Hydrogel mechanical properties affect twitching speed. (i) Population mean twitching speed increases with Young’s modulus but with limited correlation (Pearson correlation coefficient *r *= 0.69). (ii) Closeup of twitching speed as a function of modulus at low stiffness. Despite modulus being nearly identical on the four gels, we found large differences in twitching speeds. (E) Twitching speed decreases with increasing hydrogel mesh size, with relatively stronger correlation (Pearson correlation coefficient *r* = −0.81). For panels D and E, dashed lines indicate the linear fit of the data. Circles represent the mean across three biological replicates, and black bars represent standard deviation (SD). The horizontal gray line represents the mean speed across three biological replicates for a T4P retraction-deficient mutant (*ΔflicΔpilTU*), and the thickness of the line corresponds to the SD. Colored circles correspond to the gels 6 kDa, 10% (red); 0.7, 10% (blue); and 0.7 kDa, 20% (green). Black circles represent the speed values for the gels 3.4 kDa, 10% (*E *= 27 kPa, ξ = 5.9 nm); 6 kDa, 20% (*E *= 265 kPa, ξ = 5.3 nm); and 6 kDa, 20% (*E *= 470 kPa, ξ = 4.4 nm). Visualizations of biofilms grown on these gels are in Fig. S1. For panel ii in panel D, we performed a one-way ANOVA statistical test for gels with similar moduli, followed by a *post hoc* Tukey test. For 6 kDa, 10% and 3.4 kDa, 10%, we could not resolve statistically significant differences in twitching speed compared to a T4P retraction-deficient mutant (*ΔflicΔpilTU*, gray line). The differences between 6 kDa, 10% and 0.7 kDa, 10% (*P < *0.001) and between 2 kDa, 10% and 0.7 kDa, 10% (*P < *0.01) are statistically significant. All the data relative to twitching trajectories and speed presented in this figure were obtained using a *ΔfliC* mutant. The numerical values can be found in Table S3 at https://doi.org/10.5281/zenodo.7585216.

To further explore this hypothesis, we performed extensive measurements of P. aeruginosa twitching at the single-cell level on different PEGDA hydrogels. We recorded the trajectories of hundreds of cells per condition for a *ΔfliC* mutant and computed mean speed for each cell along their track (Fig. 2A to C; Movie S3). Single P. aeruginosa cells barely migrated on PEGDA hydrogels that favored the formation of defined dome-like biofilms (Fig. 2A, panel i, and Fig. 2C). In contrast, cells were much more motile on hydrogels that favored the formation of biofilm monolayers (Fig. 2A, panel ii, and Fig. 2C). Thus, the final architecture of a biofilm reflects the motility patterns observed on the distinct hydrogels. On substrates inhibiting motility, P. aeruginosa cells divide and accumulate near the initial founder cells, forming tightly packed dome-shaped biofilms. On substrates promoting motility, cells disperse on the surface as they divide, thereby limiting accumulation near founder cells but promoting surface occupation.

10.1128/mbio.03518-22.9 MOVIE S3 Time-lapse visualization of P. aeruginosa cells twitching on PEGDA hydrogels (6 kDa, 10% [wt/vol] on the left; 0.7 kDa, 20% [wt/wt] on the right). Download Movie S3, AVI file, 8.2 MB. Copyright © 2023 Cont et al. 2023 Cont et al. https://creativecommons.org/licenses/by/4.0/ This content is distributed under the terms of the Creative Commons Attribution 4.0 International license.

How does the material substrate modulate twitching motility? Consistent with our initial qualitative observations of biofilm architecture (Fig. 1B), we found only a slight correlation between PEGDA Young’s modulus and twitching speed (Fig. 2D, panel i). At equal polymer chain length, increasing stiffness with larger precursor concentration sped up twitching (visible for gels made from precursors with molecular weights of 6 and 0.7 kDa). However, we found a subset of data in which this trend failed across gels with same concentration but different chain lengths: we measured a 3-fold change in twitching speed on hydrogels of nearly identical moduli (Fig. 2D, panel ii). We therefore wondered how bacteria could perceive these materials at their scale. Given the importance of cell and T4P attachment in twitching motility, we reasoned that hydrogel surface density and topology may affect P. aeruginosa’s motility. To estimate substrate density at the surface, we measured the mesh size of the hydrogel films using equilibrium swelling theory. Twitching speeds showed a stronger, negative correlation with mesh size across concentrations and chain lengths (Fig. 2E). This revealed that hydrogel mesh size has an impact on twitching motility of single P. aeruginosa cells. Mesh may affect motility by affecting cell body adhesion or efficiency of T4P force generation. Substrates stiffness had been shown to affect bacterial adhesion (21). We therefore verified whether P. aeruginosa initial cell density was different between gels. However, we could not distinguish any difference in initial bacterial density across conditions, ruling out the possibility that initial adhesion causes the different biofilm morphologies (Fig. S2A). We then explored the possibility that differences in adhesion strength of the cell body might explain changes in twitching speed. To test this hypothesis, we applied hydrodynamic forces on hydrogel-associated P. aeruginosa Δ*fliCΔpilA*. We measured bacterial detachment at 0.2 and 2 Pa mean shear stress. We found that the strength of adhesion of bacterial cell bodies is indistinguishable between the three hydrogels (Fig. S2B, panel i). There was also no difference in bacterial adhesion strength in Δ*fliC* on the different hydrogel compositions (Fig. S2B, panel ii). The surface motility of WT also increased as a function of mesh size, demonstrating that the flagellum does not play a role in mechanical control of twitching (Fig. S3A).

10.1128/mbio.03518-22.2 FIG S2 P. aeruginosa initial attachment and adhesion strength are independent of hydrogel mesh size. (A) Quantification of the number of P. aeruginosa attached to hydrogels before twitching and biofilm experiments for (i) Δ*fliC* and (ii) pilus-deficient mutant Δ*fliCΔpil*A. There is no detectable difference in bacterial attachment between gel compositions. (B) Percentage of cells that remain attached after applying a shear stress of 0.2 or 2 Pa for a T4P-deficient mutant Δ*fliCΔpilA* (i) and Δ*fliC* (ii). The cells are uniformly removed by the flow, independently of hydrogel mesh size. Circles represent biological replicates, and black bars represent means and SD of displayed values. The statistics in both panels A and B show that the one-way ANOVA statistical test did not reject the null hypothesis; the means are therefore not significantly different. The numerical values can be found in Table S6 at https://doi.org/10.5281/zenodo.7585216. Download FIG S2, PNG file, 0.5 MB. Copyright © 2023 Cont et al. 2023 Cont et al. https://creativecommons.org/licenses/by/4.0/ This content is distributed under the terms of the Creative Commons Attribution 4.0 International license.

10.1128/mbio.03518-22.3 FIG S3 Flagella and Pel are not affecting the mechanoregulation of twitching motility. (A) (i) Comparison of twitching speed on the different gel compositions for WT PAO1 and PAO1 *ΔfliC*. Black circles represent the mean across three biological replicates, and black bars represent standard deviation (SD). Red circles represent one biological replicate. (ii) Trajectories of 300 randomly selected PAO1 cells on four different hydrogels. Twitching speed values for one replicate are indicated underneath. (B) Trajectories of 200 randomly selected PAO1 *Δpel* cells on three different hydrogels. Twitching speed values for one replicate are indicated underneath. Download FIG S3, PNG file, 0.4 MB. Copyright © 2023 Cont et al. 2023 Cont et al. https://creativecommons.org/licenses/by/4.0/ This content is distributed under the terms of the Creative Commons Attribution 4.0 International license.

As exopolysaccharide (EPS) production participates in initial P. aeruginosa surface exploration by chemically patterning the surface to generate trails (26), we also tested mutants that cannot produce Psl or Pel polysaccharides. *Δpsl* mutant could not adhere to the gel surfaces so that we could not quantify twitching. The *Δpel* mutant could, however, attach and twitch. The twitching speed of single *Δpel* mutant cells increases with decreasing mesh size, as does WT (Fig. S3B). These results show that Psl is necessary at least for attachment to the soft surface, but Pel is not required. In addition, we explored the alternative hypothesis that material stiffness could differentially stimulate mechanosensing that transcriptionally regulates adhesion and motility. For example, c-di-GMP levels increase on surface over the time scale of hours to regulate the production of EPS matrix (23). In addition, material stiffness regulates cAMP levels upon surface contact via T4Ps (29). To test the contributions of mechanosensing in early surface exploration, we measured changes in intracellular levels of cAMP and c-di-GMP using the fluorescent transcriptional reporters for *PaQa-yfp* and P*_cdra_-gfp*, respectively. We could not detect any difference in intensity of these reporters across gel compositions on the time scale of our twitching experiments, ruling out the mechanosensing hypothesis (Fig. S4).

10.1128/mbio.03518-22.4 FIG S4 P. aeruginosa does not increase intracellular levels of cAMP and c-di-GMP on hydrogels of different compositions during early times of surface colonization. (A) cAMP levels measured by PaQa-YFP reporter fluorescence. (B) c-di-GMP levels measured by cdrA-GFP reporter fluorescence. The cells were imaged after 30 and 90 minutes of contact with the surface. Circles represent biological replicates, and black bars represent means and SD of displayed values. One-way ANOVA statistical test did not reject the null hypothesis; the means are therefore not significantly different. The numerical values are in Table S7 at https://doi.org/10.5281/zenodo.7585216. Download FIG S4, PNG file, 0.3 MB. Copyright © 2023 Cont et al. 2023 Cont et al. https://creativecommons.org/licenses/by/4.0/ This content is distributed under the terms of the Creative Commons Attribution 4.0 International license.

As a result, we propose a model in which the likelihood of T4P attachment depends on mesh size rather than the strength of cell body adhesion or the activation of mechanosensory systems. The differences in T4P attachment ultimately controls the rate of productive T4P retractions. Consistent with this model, twitching speeds increase on hydrogels with mesh sizes below 5 nm, a dimension that corresponds to the diameter of the T4P fiber (30). Overall, our results suggest that T4P attachment to the hydrogel substrate with larger mesh sizes is less frequent, limiting the efficiency of force transmission during retraction.

### Substrate physical properties affect antibiotic tolerance of biofilms.

The biofilm lifestyle is a major contributor of human chronic infections due to its resilience against antibiotic treatments (5, 7, 31). Chronically infected patients are subject to lifelong P. aeruginosa infection even under strong antibiotic therapy, which favors the emergence of antibiotic-resistant mutants. Multiple bacterial physiological factors improve tolerance to antibiotics. In biofilms, matrix impermeability, metabolic state of residents, and increased cell density all contribute to protecting single bacteria from antibiotic stress (5, 7, 31). However, we know very little about how environmental factors influence the sensitivity of P. aeruginosa to antibiotics by regulating biofilm formation. In light of the distinct biofilm architectures observed on PEGDA hydrogels, we hypothesized that antibiotic efficacy could differ as a result of material properties. We thus tested the efficacy of colistin, a last-resort antibiotic against P. aeruginosa infections.

**FIG 3:**
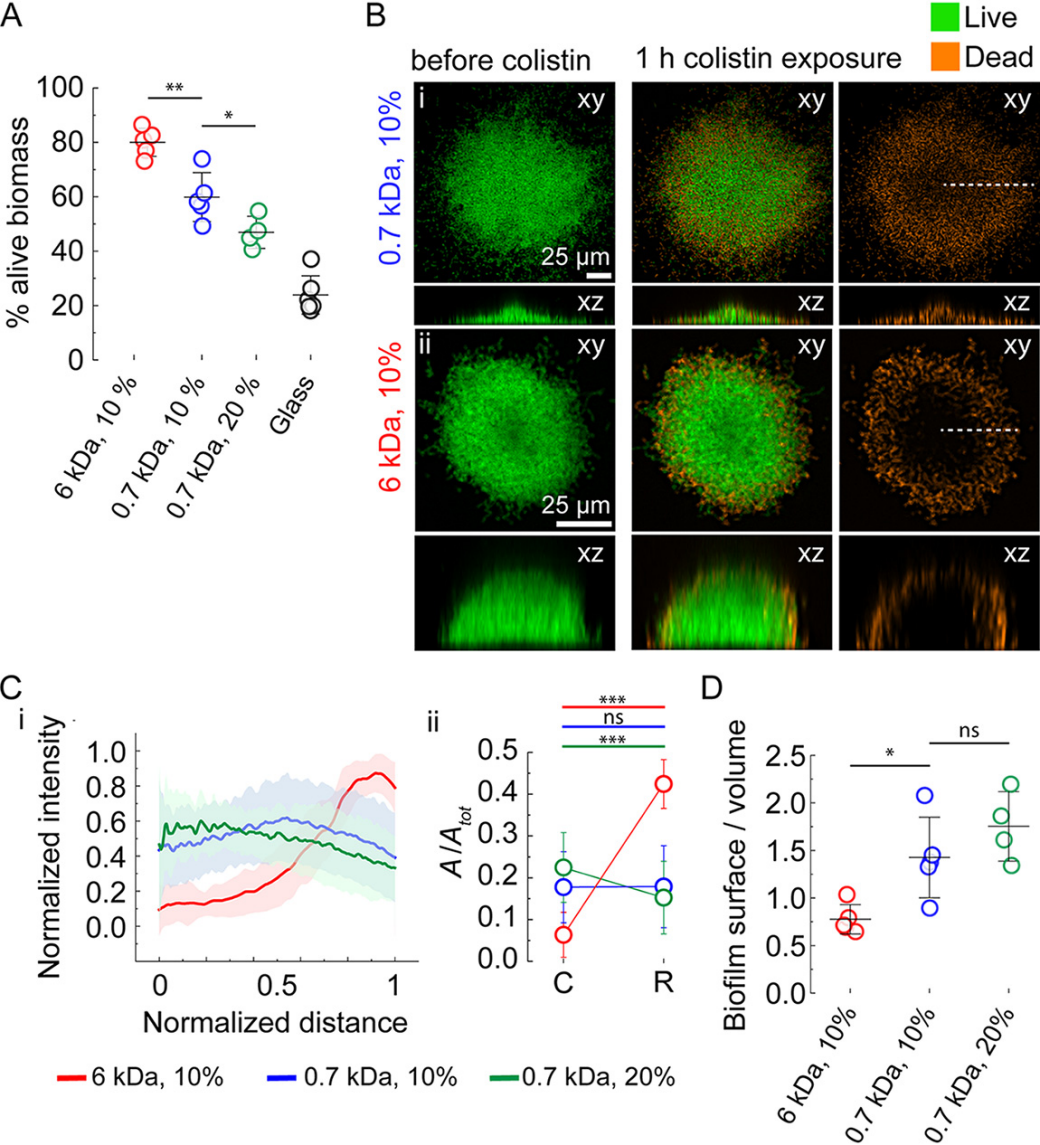
Physical control of biofilm architecture promotes P. aeruginosa’s tolerance to antibiotics. (A) Quantification of live biomass after a 1-h colistin treatment, relative to the total biomass before antibiotic exposure. Biofilms grown on hydrogels with larger mesh size are more tolerant of colistin. (B) In-plane and cross-sectional confocal visualizations shows differences in colistin-killing patterns. (C) (i) Dead stain fluorescence intensity profiles computed along a biofilm radius (dashed lines in B) highlight a more uniform distribution of dead cells in biofilms grown on hydrogels with smaller mesh size. Distance is normalized to each biofilm radius, where 0 indicates the center of the biofilm, and 1 indicates the periphery. Lines represent the average across around 30 single colonies selected from stacks acquired for three biological replicates, and the shaded area corresponds to the standard deviation. (ii) Integrated normalized area under the curves in the biofilm core (*C*, between 0 and 0.2 distance unit) and the biofilm rim (*R*, between 0.8 and 1 distance unit). Circles represent the integrated normalized area, and error bars represent the SD from the curve shown in panel i. (D) Surface-to-volume ratios for biofilms grown on different gels. Lower surface-to-volume ratios decrease the overall exposure of single cells to antibiotics, which represses bacterial killing and increases tolerance to colistin. For panels A and D, circles represent the mean value for each chip (four to five chips distributed across three biological replicates), black bars represent means, and error bars represent the SD across these values. The statistics in panels A and D are from one-way ANOVA, followed by a *post hoc* Tukey test if the null hypothesis was rejected. In panel A, the differences between 6 kDa, 10% and 0.7 kDa, 10% (*P < *0.01) and between 0.7 kDa, 10% and 0.7 kDa, 20% (*P < *0.05) are statistically significant. In panel D, the difference between 6 kDa, 10% and 0.7 kDa, 10% (*P < *0.05) is statistically significant. The statistics in panel ii in panel C are paired samples Student’s *t* test. ***, *P < *0.001. The numerical values can be found in Table S4 at https://doi.org/10.5281/zenodo.7585216. ns, not significant.

We grew biofilms on the different hydrogels for 46 h and subsequently challenged them with colistin for 1 h. To test antibiotic efficacy, we measured the volume of live biomass after treatment. After 1 h of colistin treatment, 50% of the population was still alive for biofilms growing on smaller-mesh-size hydrogels (Fig. 3A). This proportion increased to 60% on intermediate hydrogels and went as high as 80% on the larger-mesh-size hydrogels, highlighting a strong decrease in drug efficacy. We compared these results with biofilms grown on glass, in which cells twitch with an average speed of 0.52 ± 0.02 μm/min. In this case, after treatment with colistin, only 25% of the population survives. High-resolution confocal images of biofilms stained with propidium iodide to highlight cell death revealed distinct spatial patterns of colistin killing (Fig. 3B). On small-pore-size gels, bacteria were killed uniformly, irrespective of their position in the biofilm. In contrast, biofilms grown on larger-mesh-size hydrogels showed heterogeneity in cell death. Intensity profiles of biofilms stained with propidium iodide show that on these gels, bacteria at the outer edge of colonies (rim [R]) were mostly dead, while cells at the core (C) of biofilms remained largely unaffected by colistin treatment (Fig. 3C). Given the spatial pattern of killing, we reasoned that such differences were due to colistin transport into the biofilm. We therefore computed the surface-to-volume ratio in each architecture. Defined biofilms growing on more porous gels have lower surface-to-volume ratio than less porous gels (Fig. 3D). Biofilms of low surface-to-volume ratio growing on large-mesh-size gels are densest (Fig. S5A). Thus, the cells at the biofilm core are protected by the ones at the rim. In contrast, flat biofilms that have high surface-to-volume ratio are less dense, thereby exposing single cells to the external fluidic environment. Thus, biofilms growing on more porous gels are more tolerant due to longer diffusion times toward the biofilm core. Treatment on the less porous gels is more efficient as single cells are further exposed to the surrounding fluid. To visualize antibiotic penetration, we treated biofilms grown on large-mesh-size hydrogels with fluorescently labeled polymyxin B. Consistent with our hypothesis, the concentration of the antibiotic in the biofilm core was lower than the periphery (Fig. S5B). However, we cannot exclude that additional other factors such as metabolic heterogeneity affect the susceptibility of cells in the core of the biofilm.

10.1128/mbio.03518-22.5 FIG S5 Hydrogel physical properties affect cell density of biofilms and antibiotic penetration. (A) (i) Biofilms are more dense on hydrogels with larger mesh size. The biofilm density is defined as the percentage of occupied area within each colony. Each circle corresponds to one colony, and black bars represent means and standard deviation across all values. Colonies were selected from stacks acquired for three biological replicates. The statistics were from one-way ANOVA, followed by a *post hoc* Tukey test if the null hypothesis was rejected. Differences between 6 kDa, 10% and 0.7 kDa, 10% (*P < *0.001) and between 0.7 kDa, 10% and 0.7 kDa, 20% (*P < *0.01) are significant. (ii) Frequency distribution of biofilm surface density on the different gels. On gels with smaller mesh size the frequency of high surface density areas decreases, while the frequency of lower surface density areas increases. Lines represent the kernel density estimate (KDE) of the distributions. The numerical values are in Table S8 at https://doi.org/10.5281/zenodo.7585216. (B) (i) In-plane and cross-sectional confocal visualizations of biofilms grown on 6 kDa, 10% before and after exposure with rhodamine B-labeled polymyxin B. (ii) Rhodamine B-labeled polymyxin B fluorescence intensity profiles computed along a biofilm radius highlight a lower intensity in the biofilm core. Distance is normalized to each biofilm radius. The lines represent the average across 12 single colonies selected from stacks acquired for 1 biological replicate, and the shaded areas correspond to the standard deviation. Download FIG S5, PNG file, 0.5 MB. Copyright © 2023 Cont et al. 2023 Cont et al. https://creativecommons.org/licenses/by/4.0/ This content is distributed under the terms of the Creative Commons Attribution 4.0 International license.

### Material physical properties mediate biofilm heterogeneity.

**FIG 4:**
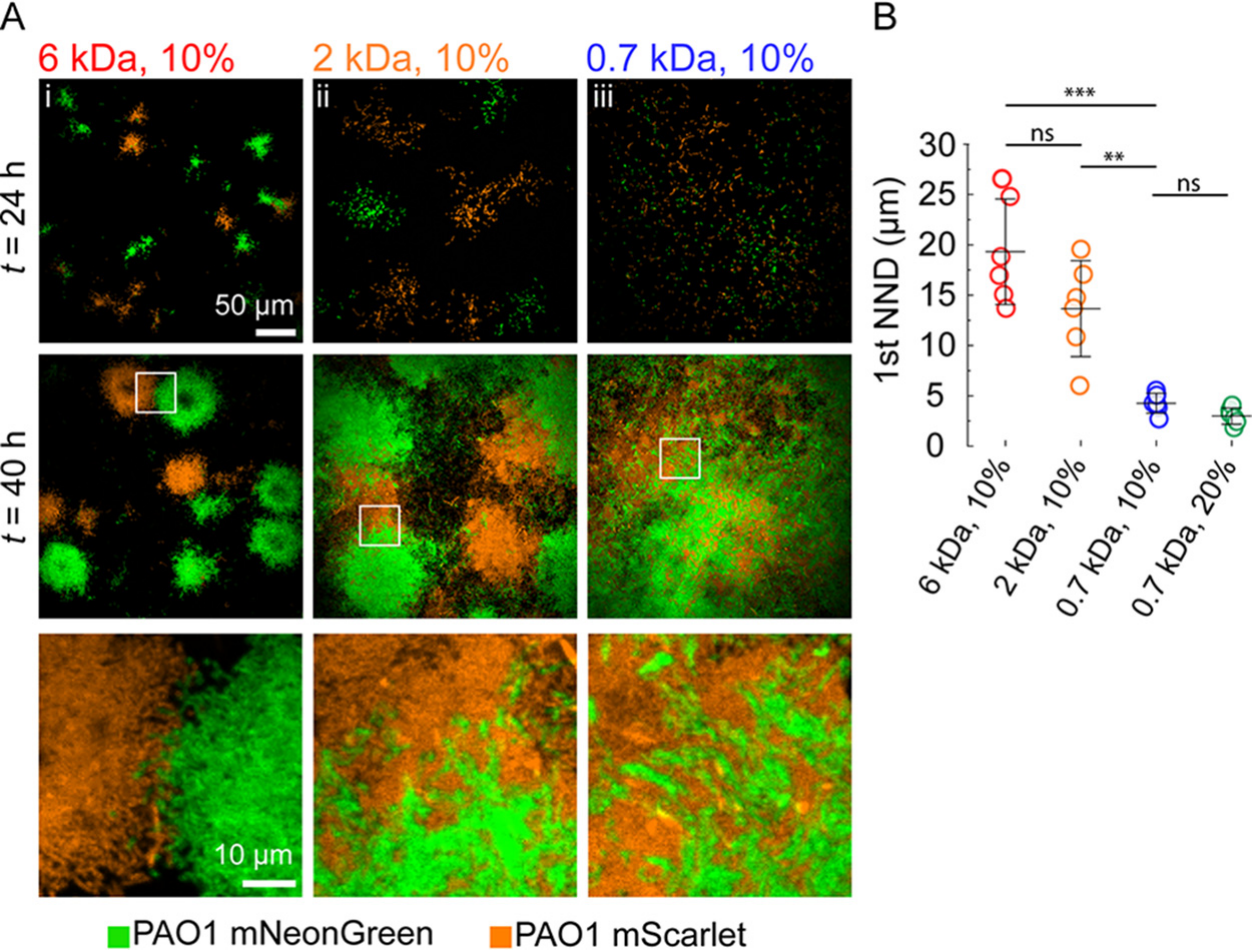
Hydrogel substrates regulate the spatial organization of heterogeneous P. aeruginosa biofilms. We grew biofilms from a mixture of P. aeruginosa clones constitutively expressing mScarlet (orange) or mNeonGreen (green). (A) In-plane confocal visualization at 24 and 40 h of heterogeneous biofilms on hydrogels with identical modulus but different mesh sizes. Larger mesh size promotes the growth of segregated biofilm clusters. In contrast, hydrogels with smaller mesh sizes promotes clonal mixing due to increased surface exploration. The bottom row shows close up views from the regions indicated by white frames. (B) Quantification of the mean first nearest neighbor distance (1st NND) between the mNeonGreen and mScarlet clones. Bacteria forming biofilms on small-mesh-size hydrogels are more prone to encounters with different clones, forcing them to compete. Circles represent the mean value for each chip (two chips for each biological triplicate, six in total), black bars represent their means, and error bars represent their SD. The statistics are from one-way ANOVA, followed by a *post hoc* Tukey test if the null hypothesis was rejected. Differences between 6 kDa, 10% and 0.7 kDa, 10% (*P < *0.001) and between 2 kDa, 10% and 0.7 kDa, 10% (*P < *0.01) are statistically significant. The numerical values are shown in Table S5 at https://doi.org/10.5281/zenodo.7585216.

Initial patterns of surface colonization are crucial to the architecture of biofilms. These patterns can also control the foundations of bacterial lineages, thereby influencing the interactions between different bacterial strains colonizing the surface evolution of microbial interaction traits. The mechanisms by which environmental conditions, such as fluid flow, and microbial response to these factors influence the spatial architecture of polymicrobial communities, however, are still unclear. Single-cell movements modulate the spatial organization of heterogeneous biofilms, ultimately governing how different clones or species compete or cooperate (32, 33). In flow, swimming motility tends to disperse Caulobacter crescentus biofilm lineages by spreading out the progeny of founder cells (34). This in turn affects the mixing of different clones coming from distinct founder cells. By analogy, we reasoned that twitching patterns observed on the different materials may affect the clonal organization by affecting lineage structure. We therefore explored the relationship between hydrogel mechanical properties and the mixing of heterogeneous biofilms. We grew biofilms from mixtures of two wild-type P. aeruginosa strains that each constitutively expressed the fluorescent proteins mScarlet and mNeonGreen (Fig. 4A). On large-mesh-size hydrogels that inhibited motility, biofilms formed into separate, isolated clusters (Fig. 4A, panel i). Finding P. aeruginosa cells of one color within a biofilm of the other was rare. mScarlet- and mNeonGreen-expressing cells were found only in close proximity when biofilms of distinct clones grew sufficiently to touch each other. Clonal lineages became less segregated, however, as hydrogel mesh size decreased, permitting twitching-dependent dispersion. On intermediate-mesh-size gels, while biofilms grew into defined colonies, there was a clear mixing between clones (Fig. 4A, panel ii; Fig. S6, panel i). Finally, on the hydrogels with smallest mesh size, clones were well mixed (Fig. 4A, panel iii; Fig. S6, panel ii). For each hydrogel condition, we computed the mean first nearest neighbor distance between the mNeonGreen and mScarlet clones (Fig. 4B). This distance decreased from 20 μm on large-mesh-size hydrogels (a length-scale corresponding to the typical radius of a biofilm) to 3 μm on the lowest-mesh-size hydrogel (corresponding to the size of a P. aeruginosa cell). Altogether, we showed that substrate material can have a strong influence on the distribution of genetically distinct bacterial population on the surface. The material-dependent mixing is of key importance in the fitness of each clone and the evolution of traits that mediate interactions.

10.1128/mbio.03518-22.6 FIG S6 Hydrogel substrates regulate the spatial organization of heterogeneous P. aeruginosa biofilms. In-plane confocal visualization at 40 h of heterogeneous biofilms on hydrogels with different mesh sizes and modulus. Biofilms appear more mixed on hydrogels with smaller mesh size and bigger modulus. For 6 kDa, 20%, *E *= 265 kPa and ξ = 5.3 nm; for 6 kDa, 20%, *E *= 470 kPa and ξ = 4.4 nm. The biofilm mixing is qualitatively similar to the one on hydrogels with comparable twitching speed (6 kDa, 20% with 2 kDa, 10% and 6 kDa, 30% with 0.7 kDa, 10%). Download FIG S6, PNG file, 6.4 MB. Copyright © 2023 Cont et al. 2023 Cont et al. https://creativecommons.org/licenses/by/4.0/ This content is distributed under the terms of the Creative Commons Attribution 4.0 International license.

## DISCUSSION

Although mechanics play a role in P. aeruginosa surface adaptation, how these communities colonize physically realistic environments has been overlooked (6). In particular, we know little about how mechanical signals regulate biofilm formation and how biofilms form on soft tissue-like materials (10). Here, by investigating the regulation of biofilm architecture at the surface of defined and tunable hydrogels, we found that material physical properties can determine biofilm architecture by influencing the mechanical activity of T4Ps during twitching motility of single P. aeruginosa cells.

Theory predicts that twitching migration speed increases with the rigidity of continuous materials in regimes in which adhesion of the cell body is weak compared to T4Ps (28, 35). On hydrogels, we observe that twitching speed depends on cross-linking density rather than on Young’s modulus. The discrepancy between theory and experiments points out to the way bacteria perceive their mechanical environments. On hydrogels, pore size could be sufficiently large so that T4Ps attach with lower frequency or strength. As such, P. aeruginosa does not experience a continuous material. Consistent with this model, we found that cells start moving on hydrogels with a mesh size below 5 nm, a value that corresponds to the width of the pilus (30). In summary, we propose a model in which twitching speed is determined by the probability of T4Ps to attach to the material surface. Thus, fewer binding events would limit the frequency of productive T4P retractions. We also measured that increasing the stiffness of the hydrogels by modulating the concentration of the precursors promotes twitching motility. These results are consistent with recent studies of P. aeruginosa on polyacrylamide gels (35). By extension, mechanics could regulate the architecture and antibiotic tolerance of biofilms of other piliated species. Looking further, mechanisms involving fimbrae, flagella, or other protein receptors in the initial steps of biofilm formation may be affected by material density.

Our work provides a perspective on how bacteria touch and perceive solid materials. Force transmission and successful motility depends on T4P and cell body attachment. In this process, cells may in concert engage mechanosensory systems, such as the ones regulating the production of second messenger molecules. For example, surface contact promotes the production of cyclic di-GMP, a known regulator of biofilm formation (23). Also, T4Ps couple with a chemotaxis-like system called Chp to guide twitching motility and upregulate virulence factors upon surface association (22, 36). The mechanical properties of materials substrate, more specifically substrate stiffness, can differentially stimulate c-di-GMP levels on longer time scales of biofilm formation (37, 38). In addition, a combination of theory and experiments suggests that substrate stiffness regulates cAMP levels upon surface contact in a T4P-dependent manner (29). However, we found that cAMP and c-di-GMP levels are identical across conditions during the time scale of our twitching experiments. Therefore, P. aeruginosa does not need to engage transcriptional mechanoregulation of twitching motility to generate biofilms with distinct morphologies. To fully comprehend how bacteria mechanically experience solid materials, it will be critical to address the fundamental principles of force transmission between bacteria and surfaces, identifying the mechanical regimes in which these forces actively signal to dedicated sensory systems.

By influencing the foundations of nascent microcolonies, material properties regulate biofilm morphogenesis. On hydrogels with smaller mesh size, shallow biofilms have a larger surface-to-volume ratio, improving molecular diffusion throughout the population. Biofilms that adopt a characteristic dome architecture have decreased surface-to-volume ratio. As a result, the bacterial population at the rim of the biofilm effectively protects the core population from antibiotics, which is consequently largely unaffected by the treatment. This could in turn also favor the rise of antibiotic-resistant mutants on a longer time scale (5, 39). This regulation of tolerance via material substrate physical properties adds to the many chemical and biological facets regulating sensitivity to antimicrobials (4, 5). Our observations has therefore the potential to guide the design of antifouling materials for biomedical applications.

P. aeruginosa encounters materials of distinct or heterogeneous mesh sizes as it colonizes extracellular matrices in burn wounds or mucus with altered viscoelasticity in individuals with cystic fibrosis. Our system may capture architectural transitions found in these different natural environemnts. The impact of material properties on the spatial organization of heterogeneous biofilms shows that mechanics could also play a role in social interactions between different species, influencing the relative fitness of bacteria of distinct backgrounds on an evolutionary time scale. We expect that biofilm clones forming on materials with smaller mesh sizes would enter in competitive interactions. Conversely, bigger mesh sizes could favor a positive interactions coexistence. Further efforts to understand how biofilms form in realistic physical contexts will reveal the relative contributions of mechanics in biofilm biogenesis, evolution, and how they contribute to infection dynamics.

## MATERIALS AND METHODS

### PEG hydrogel fabrication.

**(i) Solutions.** To generate PEG hydrogels, we prepared solutions of poly(ethylene glycol) diacrylate (PEGDA) as the precursor and lithium phenyl-2,4,6-trimethylbenzoylphosphinate (LAP, Tokio Chemical Industries) as the photoinitiator in M9 minimal medium (no calcium or magnesium). Different molecular weights (6,000, 3,400, 2,000, and 700 Da) and concentrations (10 wt/vol%, 20 wt/wt%, 30 wt/wt%) of PEGDA were used for the formation of the hydrogels (see Table S1 for details), while the concentration of LAP was kept constant at 2 mM.

**(ii) Hydrogel preparation for mechanical characterization.** To measure bulk moduli and mesh sizes of PEGDA hydrogels, we prepared samples by filling cylindrical molds made of PDMS (5-mm diameter, 4-mm height) with the precursor solutions. The molds were covered with a coverslip, and hydrogels were formed by irradiating the samples in a UV transilluminator (Bio-Rad Universal Hood II) for 5 min.

**(iii) Hydrogel thin film preparation in microfluidic channels.** To obtain thin and flat hydrogel layers, a prepolymer solution was sandwiched between two coverslips. One of the coverslips (25 × 60 mm, Menzel Gläser) was cleaned with isopropanol and MilliQ water. The other coverslip (22 × 40 mm, Marienfeld) was functionalized with 3-(trimethoxysilyl)propyl methacrylate (Sigma-Aldrich) to covalently link the hydrogel to the coverslip. We immersed the coverslips for 10 min in a solution composed of 1 mL of 3-(trimethoxysilyl)propyl methacrylate and 6 mL of diluted acetic acid (1:10 glacial acetic acid:water) in 200 mL of ethanol solution 70%. These were subsequently rinsed in ethanol and dried. We then deposited a 30 μL droplet of prepolymer solution on the first coverslip and sandwiched it with the second. The assembly was then placed under the UV transilluminator for 5 min to allow cross-linking. Right after polymerization, the coverslips were separated using a scalpel, thereby exposing the hydrogel film surface.

### PEG hydrogel characterization.

**(i) Bulk modulus.** The hydrogel cylinders resulting from the polymerization in the PDMS molds were immersed in M9 overnight and tested with a rheometer (TA instruments) in compression mode, at a deformation rate of 20 μm/s. Beforehand, the diameter of the cylinders was measured with a digital caliper, while the height of the cylinder was defined as the gap distance at which the force increases. The elastic modulus corresponds to the slope of the linear fit of the stress-strain curves in the range of 15% strain. The final Young’s modulus is the average modulus of three replicates. The moduli of the fabricated hydrogels span a range between 20 and 700 kPa, which corresponds to the bulk modulus of tissues such as skin, muscle, and gut (40).

**(ii) Nanoindentation.** The hydrogel-coated coverslips were immersed in M9 medium right after polymerization. Nanoindentation experiments were performed using an atomic force microscope (AFM, MFP-3D, Asylum Research, Santa Barbara, USA). The indentation probe was prepared by attaching a silica microsphere (Kromasil, Nouryon-Separation Products, Bohus, Sweden) with a radius of *R *= 11 μm to the end of a tipless cantilever (NSC-36, Mikromash, Bulgaria) with the help of a two-component epoxy glue (UHU GmbH, Germany). The effective spring constant was calculated as *k* = *k*_0_(*L*_0_/*L*)^3^ = 10.01 N/m, where *k*_0_ is the spring constant of the bare cantilever, and *L*_0_ and *L* are the distances from the base of the cantilever to its tip and to the microsphere, respectively (41). The spring constant of the bare cantilever *k*_0_ was determined according to the Sader method before attaching the microsphere (42). After installing the probe, the laser path was adjusted to center the laser beam on the photodiode and maximize the intensity. The system was then calibrated by pressing the probe against a silicon wafer in water. The force was determined as *F = kx*, and the indentation depth was thus calculated as *d = Z − x*, where *Z* is the vertical piezo displacement, and *x* is the cantilever displacement. The contact with the gel in liquid was determined at the point where the force signal began to deviate more than 2σ from the zero-force line, with σ being the standard deviation of the signal noise (~20 to 30 pN). The approach and retraction speeds were set to 1 μm/s. The measurements were performed at 25°C ± 1°C. Forty force curves were obtained at different locations of a sample. Elastic moduli were extracted by fitting the Hertzian model to the indentation parts of the measured curves, which showed no adhesion upon the approach (43). For the curves that showed a snap-in during the approach, the Johnson-Kendall-Roberts (JKR) model was used (44).

**(iii) Mesh size.** Mesh size was estimated from the equilibrium swelling theory using protocols previously described (25, 45–48). For each hydrogel cylinder, we determined the volume and the mass in the relaxed (r), swollen (s), and dry (d) states. We measured the volume of the cylinder right after polymerization (*V*_r_) and after immersion in M9 for 24 h (*V*_s_) with a caliper. We then washed the swollen hydrogels in deionized water to remove salts and dried them overnight in the oven at 80°C. We then measured the mass of the dry network (*M*_d_) and calculated *V*_d_ as *M*_d_/ρ_PEG_, with ρ_PEG_ taken to be 1.18 g/mL. We then calculated the average molecular weight between cross-links, *M*_c_ using equation 1,

(1) $$\frac{1}{\bar{M_{\text{c}}}}=\frac{2}{\bar{M_{\text{n}}}}-\;\frac{\left(\frac{\bar{v}}{V_{1}}\right)\left[\ln\left(1-v_{2,\text{s}}\right)+v_{2,\text{s}}+\text{χ}v_{2,\text{s}}^{2}\right]}{v_{2,\text{r}}\;\left[{\left(\frac{v_{2,\text{s}}}{v_{2,\text{r}}}\right)}^{\frac{1}{3}}-\frac{1}{2}\left(\frac{v_{2,\text{s}}}{v_{2,\text{r}}}\right)\right]}$$

where $\bar{v}$ is the specific volume of the polymer (taken to be 0.93 mL/g for PEG), $V_{1}$ is the molar volume of water (18 mL/mol), χ is the polymer-solvent interaction parameter (taken to be 0.426 for PEG in water), $\bar{M_{\text{n}}}$ is the average molecular weight of the polymer before cross linking, and $v_{2,\text{r}}$ and $v_{2,\text{s}}$ are the polymer volume fractions:

$$v_{2,\text{r}}=\;\frac{V_{\text{d}}}{V_{\text{r}}}\;v_{2,\text{s}}=\;\frac{V_{\text{d}}}{V_{\text{s}}}\;$$

We finally obtained the mesh size ξ with equation 2,

(2) $$\text{ξ}=v_{2,\text{s}}^{-1/3}l\sqrt{\frac{2C_{\text{n}}\bar{M_{\text{c}}}}{M_{\text{r}}}}$$

Where l is the bond length along the polymer backbone (0.15 nm), $C_{\text{n}}$ is the Flory characteristic ratio (4 for PEG), and $M_{\text{r}}$ is the molecular weight of the repeat unit (44 g/mol).

### Assembly of hydrogel-coated coverslips with microfluidic chips.

We fabricated microfluidic chips following standard soft lithography techniques. For biofilm experiments, we designed 2-cm-long, 2-mm-wide channels in Autodesk AutoCAD and printed them on a soft plastic photomask. We then coated silicon wafers with the photoresist (SU8 2150, Microchem), with a thickness of 350 μm. The wafer was exposed to UV light through the mask and developed in propylene glycol methyl ether acetate (PGMEA) (Sigma-Aldrich) in order to produce a mold. PDMS (Sylgard 184, Dow Corning) was subsequently casted on the mold and cured at 80°C overnight. After cutting out the chips, we punched 1-mm inlet and outlet ports. We finally punched a 3-mm hole right downstream of the inlet port. This hole, after being covered with a PDMS piece, acts as a bubble trap. To fabricate channels for the twitching experiments, we followed a similar procedure, but we used a different photoresist (SU8 2025 Microchem), and we adjusted the dimensions of the channel to be 500 μm wide and 100 μm high.

The final assembled chips were obtained by placing the PDMS chips on top of the hydrogel-coated coverslips right after polymerization. This results in a reversible but sufficiently strong bond between the hydrogel and the PDMS, allowing us to use the chips under flow without leakage for several days. The channels of the chips were filled with M9 medium to keep the hydrogel hydrated for at least 12 h before being used.

### Bacterial strains.

The strains used in this work are listed in Table S9 at https://doi.org/10.5281/zenodo.7585216. All strains were grown in LB medium at 37°C. Overnight bacterial cultures were diluted 1:1,000 in fresh LB and grown until midexponential phase (optical density at 600 nm: 0.3 to 0.6).

### Single-cell twitching and adhesion.

Overnight bacterial cultures of PAO1 *Δflic*, PAO1 *Δflic ΔpilTU*, PAO1, PAO1 *Δpel*, and PAO1 *Δpsl* were diluted 1:1,000 in fresh LB and grown until midexponential phase (optical density [OD], 0.4 to 0.6). Bacterial cultures were diluted to reach an optical density of 0.4 for PAO1 *Δflic* and PAO1 and 0.1 for PAO1 *Δflic ΔpilTU*. For PAO1 *Δpel* and PAO1 *Δpsl*, 200 μL of bacterial cultures at OD 0.5 were centrifuged at 6,000 rpm and resuspended in 50 μL of LB to increase the number of adhering cells. We then loaded 10 μL of the bacterial culture in the small microfluidic chips (500 μm wide and 100 μm deep) assembled with either hydrogel-coated coverslips or with glass coverslips. We let the bacteria adhere for 30 min. We connected the inlet port to a disposable syringe (BD Plastipak) filled with the medium and mounted onto a syringe pump (KD Scientific), using a 1.09-mm-outer diameter polyethylene tube (Instech) and a 27-gauge needle (Instech). For twitching experiments, we used a flow of 60 μL · h^−1^ for 5 min in order to remove bacteria that did not adhere to the hydrogel surface. We then switched to a flow of 30 μL · h^−1^ before starting image acquisition. Images were taken every 30 s for 1 h.

For adhesion experiments, 200 μL of bacterial cultures of PAO1 *Δflic ΔpilA* and PAO1 *Δflic* at OD 0.5 were centrifuged at 6,000 rpm and resuspended in 50 μL of LB. We load 10 μL of the bacterial suspension in small microfluidic chips that were hold together with a custom-built clamp to avoid delamination of the chip from the gel under large flow rate. We connected the inlet port as described above, and we let the cells adhere for 30 min. We used a flow of 60 μL · h^−1^ for 5 min in order to remove bacteria that did not adhere to the hydrogel surface. Images were then taken every 10 s for 6 min in the center of the channel with a flow of 60 μL · h^−1^ for the first 2 min, 600 μL · h^−1^ for the next 2 min, and 6,000 μL · h^−1^ for the final 2 min.

We aquired images on a Nikon TiE widefield microscope equipped with a Hamamatsu ORCA Flash 4 camera. Images were acquired in phase contrast with a 20× Plan APO NA 0.75 objective.

### Biofilm formation.

Overnight bacterial cultures of PAO1 were diluted 1:1,000 in fresh LB and grown until midexponential phase. Bacterial cultures were diluted to reach an optical density of 0.05. We then loaded 6.5 μL of the diluted bacterial culture in the big channels (2 mm wide and 350 μm high) from the outlet port. It is important that injected cells do not reach the well of the bubble trap. We let the cells adhere for 30 min before starting the flow. The biofilms were grown at 25°C at a flow rate of 10 μL · min^−1^. For time-lapse visualizations of early-stage biofilm formation, we acquired images every 15 min for 15 h in phase contrast with a 40× Plan APO NA 0.9 objective. For the visualization of biofilms, we used a Nikon Eclipse Ti2-E inverted microscope coupled with a Yokogawa CSU W2 confocal spinning disk unit and equipped with a Prime 95B sCMOS camera (Photometrics). We used a 20× water immersion objective with N.A. of 0.95, and Z stacks of the biofilms were taken every 2 μm.

### Quantification of cAMP AND c-di-GMP during surface growth.

To quantify intracellular levels of cAMP, we used the PaQa-YFP reporter system as previously described (36). To quantify intracellular levels of c-di-GMP, we used the P*_cdrA_*-GFP reporter system (49). Single colonies of PAO1 containing PaQa-YFP or P*_cdrA_*-GFP reporter plasmids were grown overnight, respectively, in LB-carbenicillin and LB-gentamicin. The cultures were then diluted 1:1,000 in fresh antibiotic-free LB and grown until OD 0.5. We then loaded 10 μL of the bacterial culture in the small microfluidic chips, and we let the bacteria adhere for 25 min. We then washed the channels for 5 min and acquired images in the appropriate fluorescent channels at 30 min and 90 min. Image acquisition was done with the confocal spinning disk microscope equipped with a 100× oil immersion objective (N.A. of 1.45) as described above.

### Image processing and analysis.

Snapshot images and movies were generated with Fiji. The images were processed with macros in Fiji, and the data were analyzed in Python 3 and OriginPro.

**(i) Single-cell twitching.** When necessary, drift was corrected using the Correct 3D Drift plugin. The cells were segmented and then tracked using a Trackmate script that tracks spots based on a result table that contains the position of each cell’s center of mass in the movie. Subsequent analysis of cell trajectories was done with a custom Python script. Only tracks with a duration above 10 min were considered. Cell speed was calculated as the net displacement (distance between the last and the first spot of the track) divided by the track duration. An average speed was calculated for each replicate based on at least 50 tracks, and the displayed values are the average among three replicates and the corresponding standard deviation.

**(ii) Single-cell adhesion.** Only a portion of the stack with a width equal to half of the channel width taken in the center was considered. The cells were segmented and tracked as above. Only cells attached to the surface from time 0 were considered. We defined the shear stress at the wall of the channel as *σ*_s_ = 6 Qμ/wh^2^, where *Q* is the volumetric flow, μ is the viscosity of the fluid, and *w* and *h* are the width and the height of the channel, respectively. The initial cell number is defined as the number of cells the stays attached for at least 90 s under a shear stress of 0.02 Pa (*Q *= 60 μL · h^−1^). The percentage of cells that stays attached to the surface under a shear stress of 0.2 and 2 Pa is defined as the number of cells attached to the surface just before the next increase of shear stress normalized by the initial cell number.

**(iii) cAMP and c-di-GMP quantification.** The images were background-subtracted. The cells were then segmented, and the corresponding mean PaQa-YFP to mKate2 fluorescent intensity ratios or mean cdrA-GFP intensities were computed. For each biological replicate, at least 50 cells were analyzed. The displayed values correspond to the average intensity ratios or intensities for each biological replicate.

**(iv) Biofilm morphology.** Biofilms were imaged after 40 h of growth in proximity to the channel inlet. For each condition, we imaged 6 chips (2 chips for each biological replicate) and around 30 single colonies were selected and segmented. The radius of the colony is defined as the radius of a circle with an area equal to the substrate area covered by the biofilm. The height of the colony is defined as the maximum height of the orthogonal projection at the center of the colony (found by fitting a circle to the area covered by the biofilm). The displayed height-to-radius ratio corresponds to all measured colonies.

**(v) Biofilm spatial organization.** For mixing experiments, PAO1 mNeonGreen and PAO1 mScarlet were mixed at a 1:1 ratio before inoculation in the channels. Biofilms were imaged after 40 h of growth at the beginning of the channel. For each condition, we imaged 6 chips (2 chips for each biological replicate), and for each chip, we acquired around 10 stacks. To correct for the slide tilting, we performed a maximum intensity projection of the first three slices for each stack. Images were then segmented for the two fluorescent channels. To quantify the first nearest neighbor distance (NND), we selected 100 random pixels in the segmented mNeonGreen picture for each stack. We then calculated the distance between the selected pixels and their nearest neighbor pixels in the corresponding segmented mScarlet picture. This way we obtained an average first NND for each stack. For each chip, the highest first NND value was filtered out, and a mean value of the remaining stacks was calculated. The displayed first NND corresponds to the average first NND for each chip.

**(vi) Antibiotic treatment of biofilms.** Biofilms of PAO1 green fluorescent protein (GFP) were imaged after 46 h (time 0) of growth at the beginning of the channel. For each condition, we imaged four to five chips (distributed among three biological replicates), and for each chip, we acquired six stacks. We then switched the medium from LB to LB containing the antibiotic colistin (5 μg/mL, Acros Organics) and the dye propidium iodide (5 μM, Cayman chemical) for staining of dead cells. Biofilms were imaged every hour in the same positions set at time 0. Stacks acquired at time 0 and after antibiotic treatment were concatenated, and the drift was corrected. Similarly, to check the penetration of the antibiotic inside the biofilm, we switched the medium from LB to LB containing rhodamine B-labeled polymyxin B (5 μg/mL, Sigma-Aldrich), and the biofilms were imaged before and after 1 h exposure. To quantify the effect of the antibiotic on the biofilms, we measured the volume of live biomass (expressing GFP). Stacks were segmented, and the biofilm volume at different times was quantified with the plugin 3D Objects counter and normalized by the value at time 0. We then obtained and average value for each chip. Biofilm volume and surface at time 0 were quantified with the plugin 3D Objects counter. The substrate area covered by the biofilm was measured after performing a maximum intensity projection of the segmented stack. The exposed surface was calculated as the difference between the total surface and the substrate area covered by the biofilm. Exposed surface-to-volume ratio was calculated for each stack, and we obtained an average value for each chip.

To quantify cell death induced by colistin, we selected around 30 to 40 single colonies from stacks acquired after 1 h of antibiotic treatment. Images in the green channel were segmented and used to define the core (C) and the rim (R) of the colony by fitting a circle to the area covered by the biofilm. We then performed a radial reslice over 360° in the red channel (dead biofilm) by rotating a line with a length equal to R around one of its ends placed in C. We performed an average intensity projection of the resulting stack and measured the intensity profile along a line of length equal to R drawn slightly above the plane of contact between the biofilm and the substrate. The intensity of the curves was normalized by the highest value, while the distance was normalized by *R*. We then averaged the curves and calculated the standard deviation for each condition. We then performed integration of the curve between 0 and 0.2 (C) and between 0.8 and 1 (R), and we normalized the results by the total area under the curve. To quantify the penetration of rhodamine B-labeled polymyxin B, we performed the same operations on 12 single colonies from stacks acquired after 1 h of treatment (1 replicate), using the red channel (rhodamine B-labeled polymyxin B) for the radial reslice. The obtained average curve was smoothed by using a 40-point moving average.

**(vii) Biofilm surface density.** To quantify the density of the biofilm, we selected about 40 colonies from stacks acquired before antibiotic treatment (same colonies used for the quantification of colistin induced death). Images in the green channels were segmented and used to define the center of the colony by fitting a circle to the area covered by the biofilm. We then defined the dimension of the biofilm to be the radius of a circle that contains 90% of the total colony surface area. For each colony, we then defined a grid with the dimension of the fitting circle and made of 20 × 20-μm squares. For each square in the grid, we quantified the area fraction occupied by the biofilm on the surface. We defined the biofilm surface density as the proportion of occupied surface for each colony by averaging the values across the grid. Distributions of biofilm surface density for each gel were obtained by plotting the area fraction of all the corresponding squares.

**(viii) Statistics.** All statistical tests were run in OriginPro. For one-way analysis of variance (ANOVA) statistics, if the null hypothesis was rejected, we followed up with a *post hoc* Tukey test.
