# Supplementary figures and images for: Material Substrate Physical Properties Control Pseudomonas aeruginosa Biofilm Architecture
Source: mBio. 2023 Feb 14;14(2):e03518-22. doi: 10.1128/mbio.03518-22 (PMC10127718; doi:10.1128/mbio.03518-22)

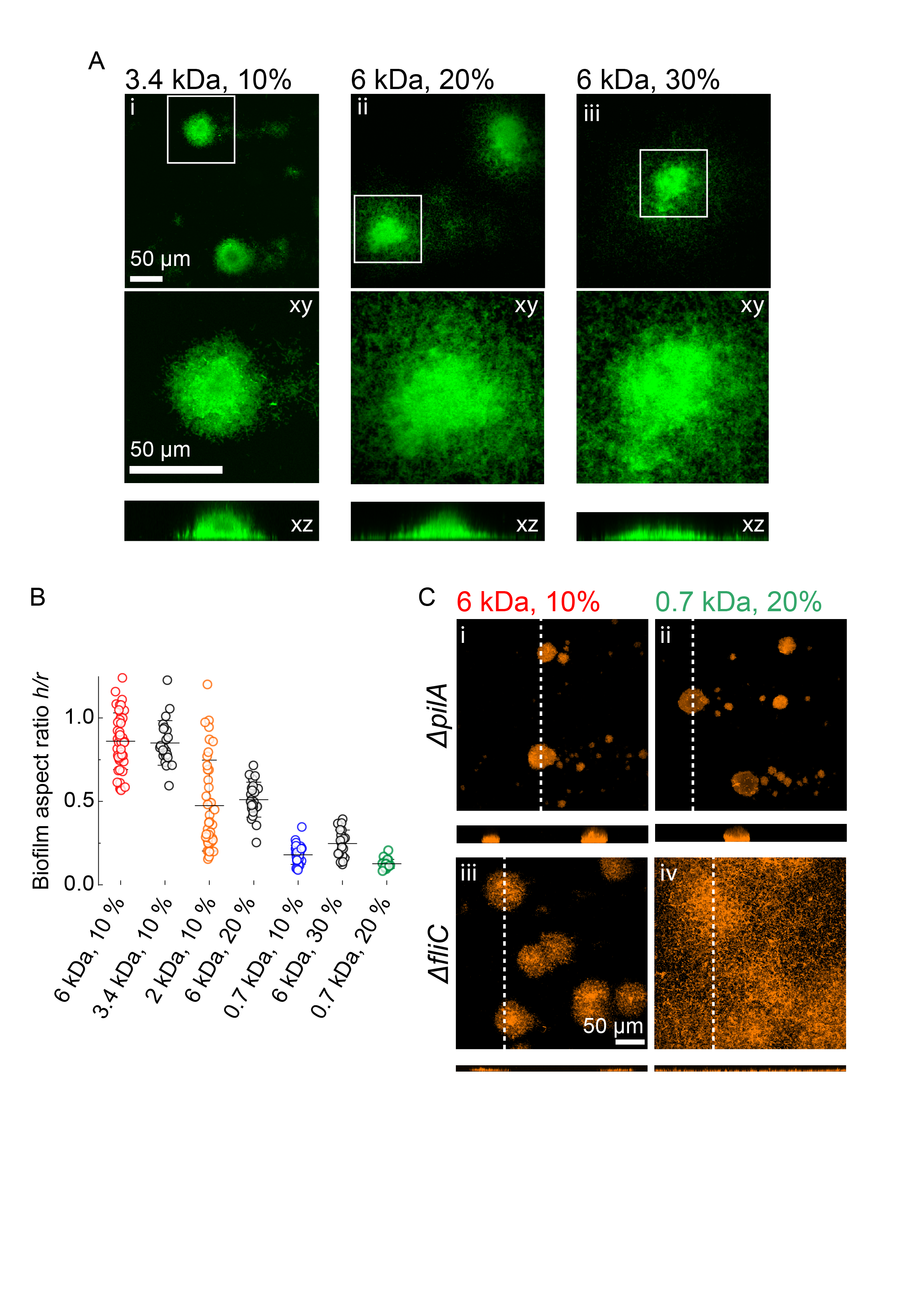

Supplement: FIG S1 [file mbio.03518-22-s0005.png]

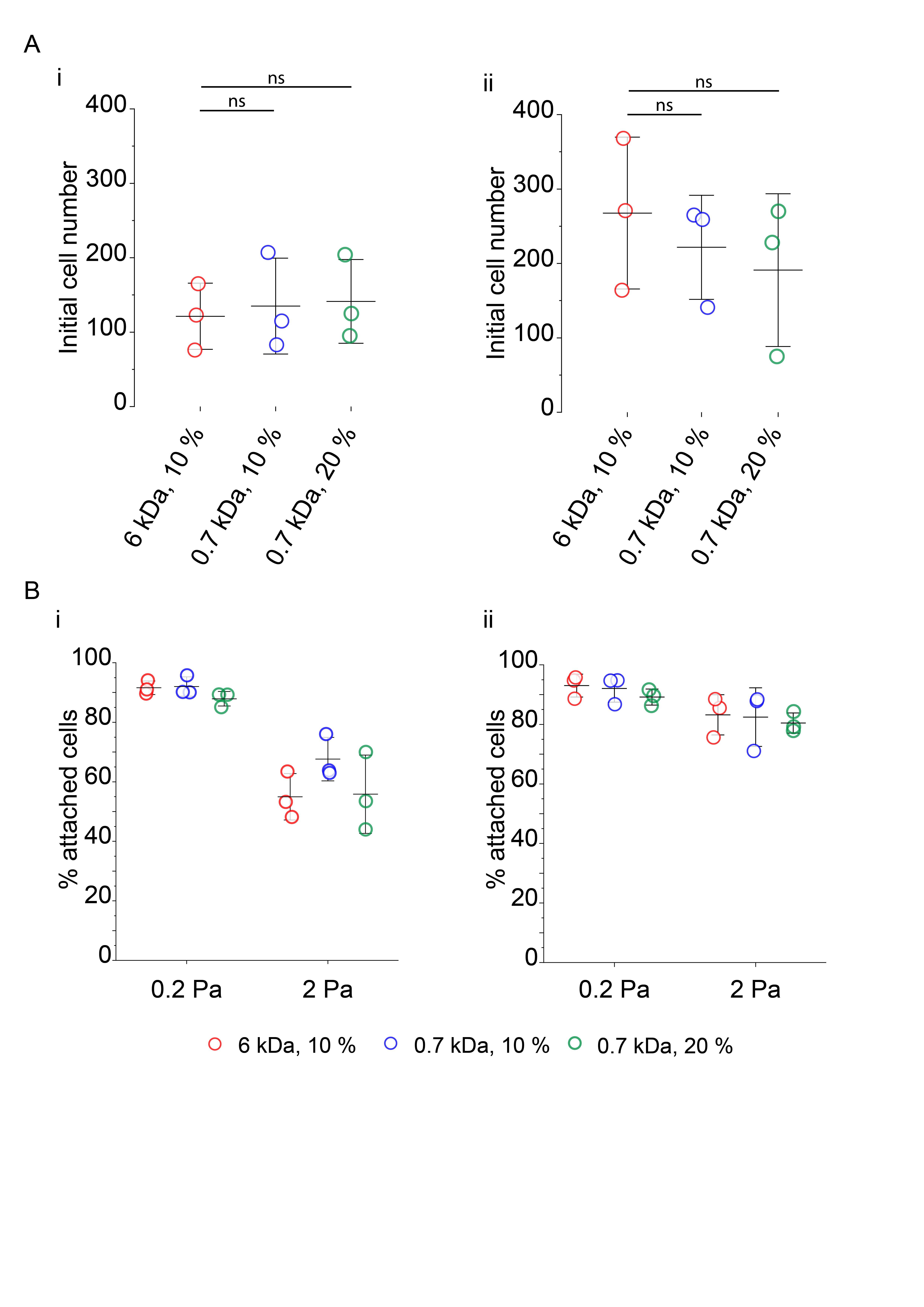

Supplement: FIG S2 [file mbio.03518-22-s0006.png]

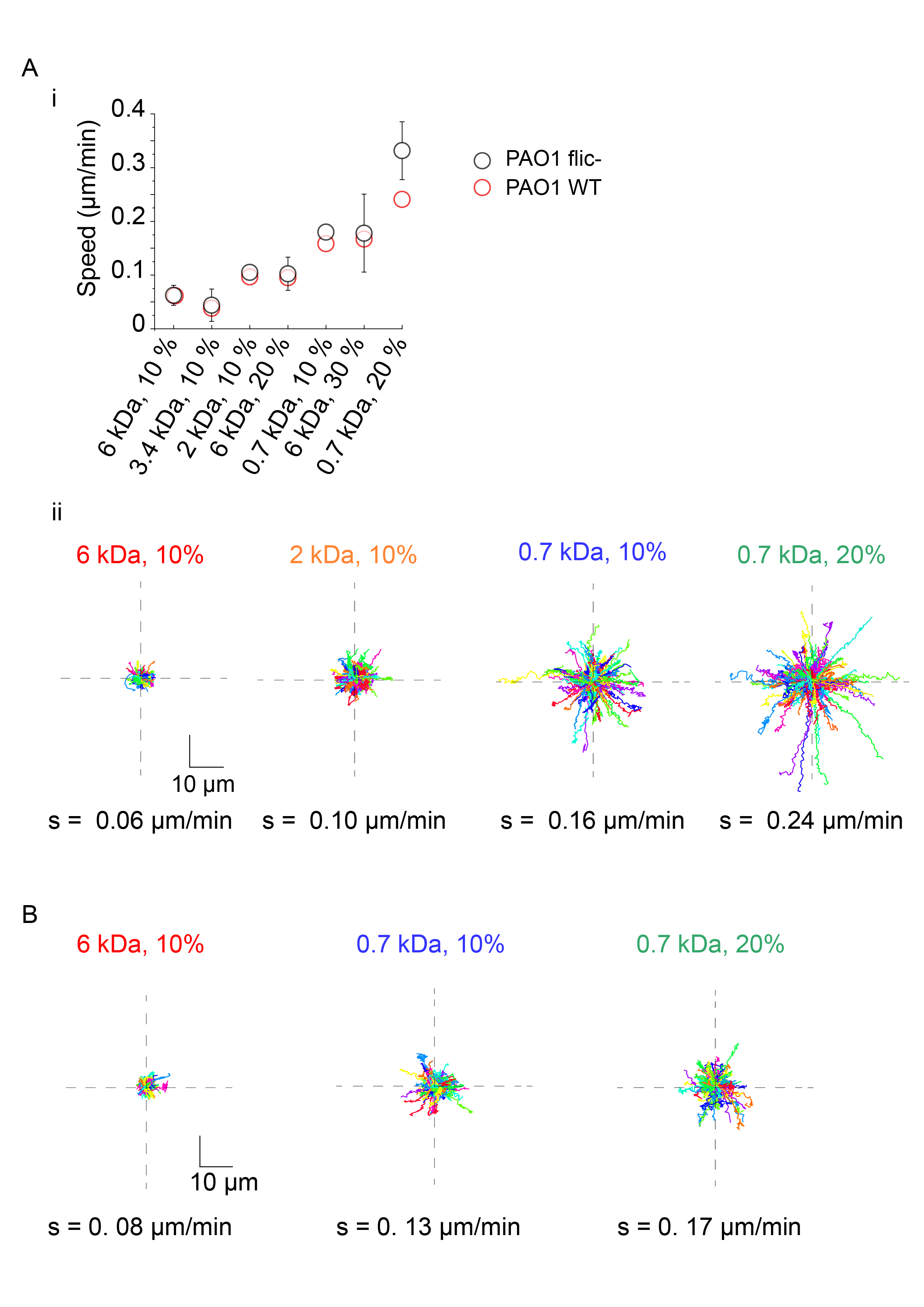

Supplement: FIG S3 [file mbio.03518-22-s0007.png]

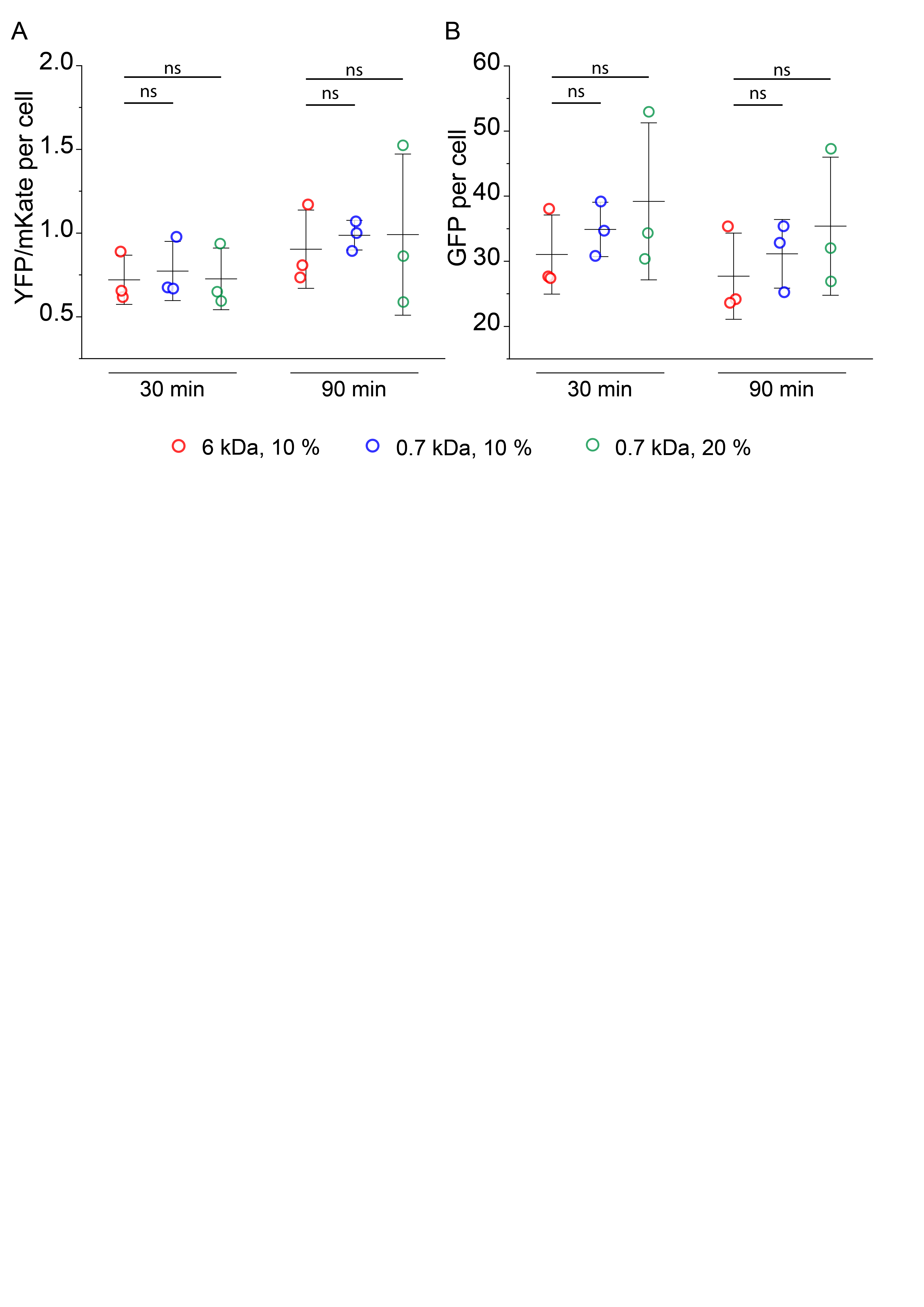

Supplement: FIG S4 [file mbio.03518-22-s0008.png]

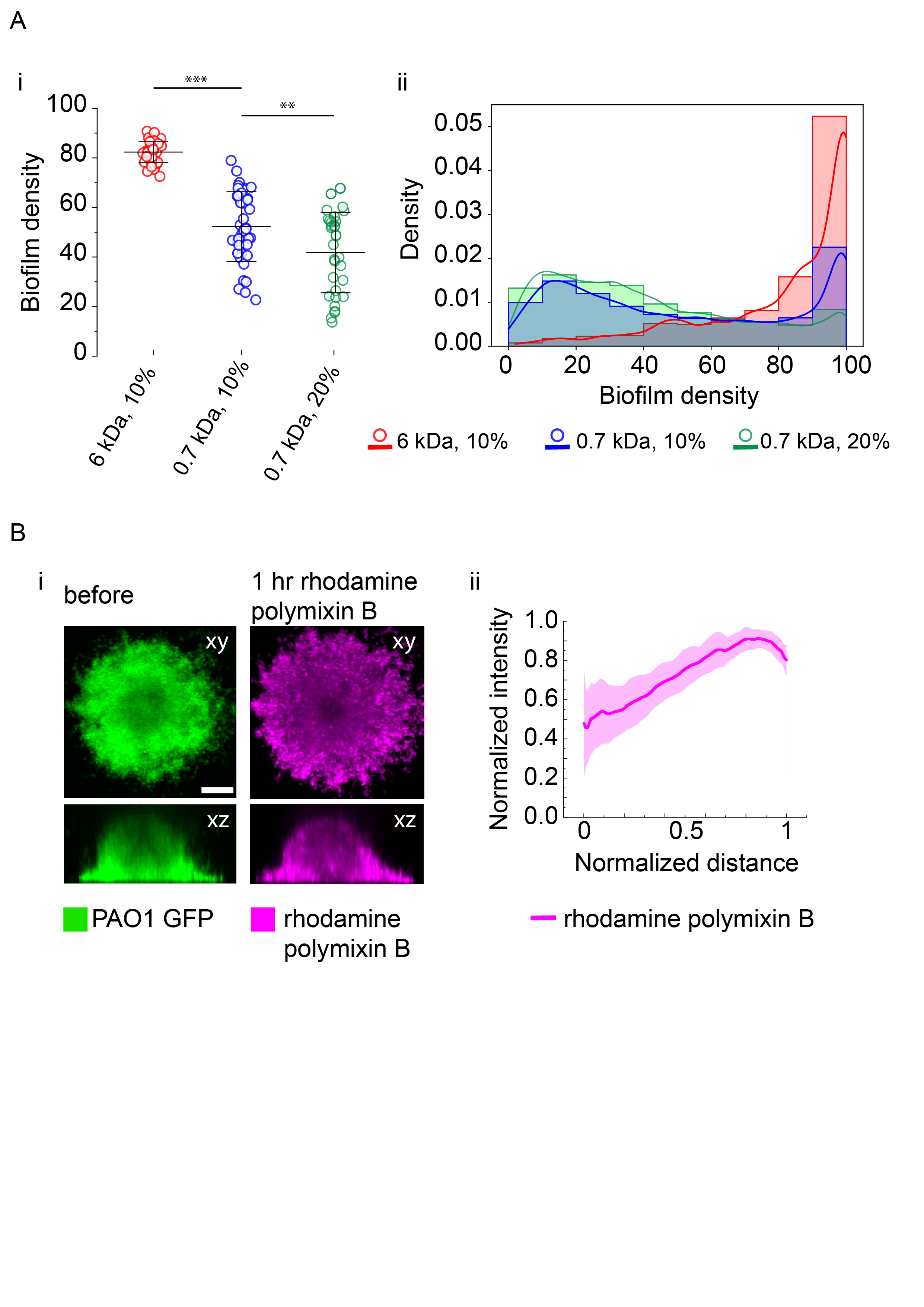

Supplement: FIG S5 [file mbio.03518-22-s0009.png]

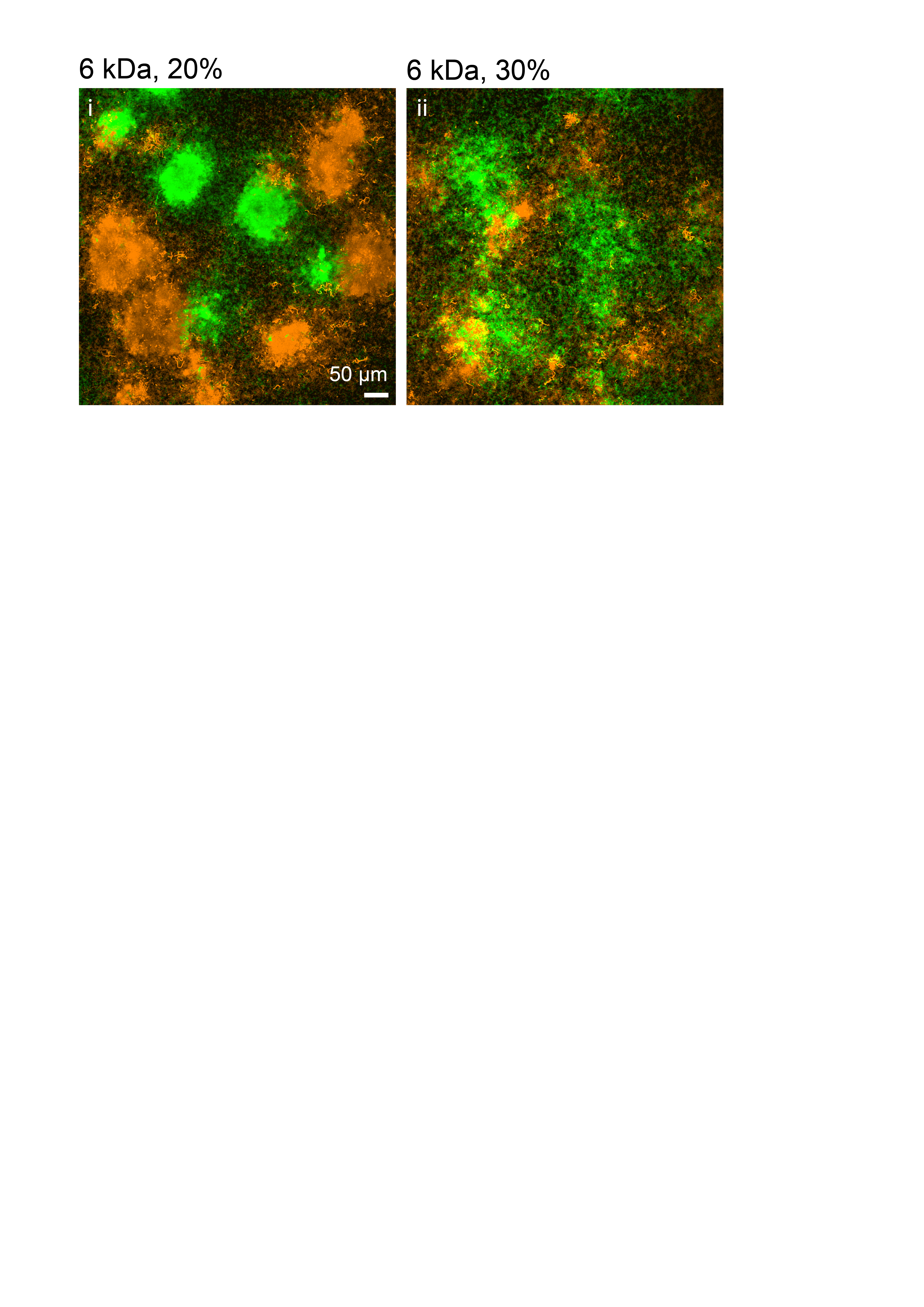

Supplement: FIG S6 [file mbio.03518-22-s0010.png]
