# Supplementary material for: Material Substrate Physical Properties Control Pseudomonas aeruginosa Biofilm Architecture
Source: mBio. 2023 Feb 14;14(2):e03518-22. doi: 10.1128/mbio.03518-22 (PMC10127718; doi:10.1128/mbio.03518-22)
Supplement: TABLE S1 [file mbio.03518-22-s0004.docx]

**Table S1: Summary of mechanical properties of PEGDA hydrogels.**

* Differences between 6 kDa, 10% and 3.4 kDa, 10% (p < 0.001), between 3.4 kDa, 10% and 2 kDa, 10% (p < 0.01), between 2 kDa, 10% and 0.7 kDa, 10% (p < 0.05) are statistically significant (one-way ANOVA, followed by a post-hoc Tukey test).

| Gel | PEGDA precursor | Concentration  (% w/v or % w/w) | *Young’s modulus* (bulk) ± SD (kPa) | *Young’s modulus* (AFM) ± SD (kPa) | Mesh size ± SD (nm) |
| --- | --- | --- | --- | --- | --- |
| 6 kDa, 10% | PEGDA MW 6000 (Biochempeg) | 10% w/v | 38.0 ± 13.9 | 33.7 ± 0.8 | 8.50 ± 1.20 * |
| 6 kDa, 20% | PEGDA MW 6000 (Biochempeg) | 20% w/w | 265.0 ± 0.3 | 218 ± 6 | 5.26 ± 0.25 |
| 6 kDa, 30% | PEGDA MW 6000 (Biochempeg) | 30% w/w | 470 ± 28 | 573 ± 27 | 4.40 ± 0.14 |
| 3.4 kDa, 10% | PEGDA MW 3400 (Biochempeg) | 10% w/v | 26.9 ± 1.1 | 26.4 ± 0.6 | 5.88 ± 0.06 * |
| 2 kDa, 10% | PEGDA MW 2000 (Biochempeg) | 10% w/v | 37.6 ± 2.1 | 26 ± 0.6 | 3.85 ± 0.17 * |
| 0.7 kDa, 10% | PEGDA MW 700 (Sigma-Aldrich) | 10% w/v | 48.0 ± 1.3 | 21.6 ± 0.6 | 2.40 ± 0.05 * |
| 0.7 kDa, 20% | PEGDA MW 700 (Sigma-Aldrich) | 20% w/w | 750 ± 57 | 486 ± 14 | 1.59 ± 0.03 |
